# Prevalence and characteristics of HPV vaccine hesitancy among parents of adolescent females across Saudi Arabia

**DOI:** 10.3389/fpubh.2024.1501358

**Published:** 2025-01-23

**Authors:** Sahar Othman, Ranya Ghamri, Wala Alhamadah, Anwaar Alsatty, Dhuha Khesfaty, Ohoud Alghamdi, Ragad Arbaein, Alaa M. Baglagel, Jumana Khayyat, Bushra Almalki

**Affiliations:** ^1^Family Medicine Department, Faculty of Medicine, King Abdulaziz University, Jeddah, Saudi Arabia; ^2^Faculty of Medicine, King Abdulaziz University, Jeddah, Saudi Arabia

**Keywords:** vaccine, adolescent, HPV, prevalence, Saudi Arabia HPV vaccine, immunization, vaccination, sexually transmitted diseases

## Abstract

**Purpose:**

Vaccination against human papillomavirus (HPV) is pivotal in preventing HPV-related diseases, including cervical cancer. However, vaccine hesitancy and their related factors among parents of adolescent females present a significant obstacle to achieving high vaccination rates. HPV vaccine hesitancy among parents of adolescents and their related factors have been studied at the regional level in Saudi Arabia, but national-level data are not available. Therefore, this study aims to assess the prevalence and characteristics of HPV vaccine hesitancy among parents of adolescent females in Saudi Arabia.

**Materials and methods:**

This cross-sectional study was conducted in Saudi Arabia among parents of adolescent females aged 11–17 years between November and December 2022. The participants completed a self-administered online questionnaire containing the validated Vaccine Hesitancy Scale (VHS), which was originally developed in 2015 by the WHO’s Strategic Advisory Group of Experts (SAGE) on immunization. Descriptive statistics, univariate binomial regression, and multivariate binary logistic regression were employed for statistical analysis.

**Results:**

Among the 667 participants who completed the questionnaire, 34% were hesitant about immunizing their adolescent daughters with the HPV vaccine. A few demographic variables were significantly associated with vaccine hesitancy, including lower household income and living in the southern region. In addition, lack of recommendation by their healthcare provider and inconvenience related to immunization with the vaccine were also associated with higher levels of hesitancy.

**Conclusion:**

HPV vaccine hesitancy remains high among parents of adolescent females in Saudi Arabia. More effort needs to be directed toward educating parents, especially during doctor’s visits, in order to increase the acceptance and vaccination rates.

## 1 Introduction

Human papilloma virus (HPV) infection is considered one of the most common sexually transmitted diseases around the world (1). This infection affects the anogenital areas and is responsible for common warts (2, 3). It has been proven that HPV infection causes cancers of the cervix, genital area, and throat (4). Vaccination against HPV is crucial for the prevention of HPV- related diseases, including cervical cancer (5, 6). However, hesitancy toward vaccination among parents of adolescent females poses a significant challenge to achieving high vaccination rates (7). Vaccine hesitancy refers to the delay in accepting a vaccine or refusal to undergo immunization with a vaccine (8). Globally, many studies have addressed vaccine hesitancy. For example, according to a survey conducted in Jimma Town, 39.02% of 369 female students in Jimma Town schools were hesitant to get the HPV vaccine (9).

Saudi Arabia, like many other countries, has implemented a comprehensive national immunization program to protect its population from vaccine-preventable diseases. With its commitment to public health measures, the Ministry of Health in Saudi Arabia has arranged vaccination programs that ensure access to various vaccines, including childhood immunization against diseases such as polio, measles, mumps, and rubella. In addition, the ministry has established an extensive network of healthcare facilities, including primary healthcare centers, hospitals, and vaccination clinics, to ensure the availability and accessibility of vaccine all around the country (10). Within this framework, the ministry has dedicated efforts toward HPV vaccination, which has now become a part of the national immunization program (11). The program recommends HPV vaccination for adolescent females aged 9 to 14 years, in accordance with the vaccination schedules recommended internationally (6). However, despite such efforts toward promoting HPV vaccination, hesitancy against this vaccine remains a challenge in Saudi Arabia (8, 12). At a national level, a previous study conducted in the Eastern Province to assess awareness and knowledge of the HPV vaccine among females and males found that only 4% of the participants had received the HPV vaccination (12, 13).

Understanding the factors influencing HPV vaccine hesitancy is very important to develop effective countermeasures and achieve good vaccination rates. Various factors have been implicated, including knowledge gaps, concerns about vaccine safety and efficacy, and the influence of misdirecting information and social media, which increase vaccine hesitancy and lower vaccine acceptance, thus affecting the achievement of high vaccination rates (5, 14). One study conducted at the Family Medicine Pediatric Clinics, King Faisal Specialist Hospital and Research Centre, Riyadh, investigated awareness and attitudes regarding the HPV vaccine among Saudi parents attending family medicine clinics in Riyadh. The study reported a poor level of awareness and attitude toward the HPV vaccine among Saudi parents. Further, those who planned on getting the vaccine seemed to have a significantly higher level of awareness of the HPV vaccine (12). Another cross-sectional study conducted among 343 parents selected randomly from the Saudi western region showed that parents had poor knowledge of HPV and its vaccine, as only 32.9% of the participating parents knew about the HPV vaccine. Moreover, the most frequent barrier for vaccination was their belief of not being at risk (75.2%) (15).

The previous studies discussed above were conducted at a local institutional level and recommended larger national studies to further examine HPV vaccine hesitancy among parents in Saudi Arabia. This study aims to fill in this research gap by assessing the prevalence and characteristics of HPV vaccine hesitancy among parents of adolescent females in Saudi Arabia. Further, another aim of this study was to determine the demographic factors that influence vaccine hesitancy.

## 2 Materials and methods

### 2.1 Study design and settings

This is a cross-sectional study conducted during the months of November and December 2022 in Saudi Arabia.

### 2.2 Participants

The study included the parents of adolescent females aged 11–17 years who willingly volunteered to participate in the study and demonstrated proficiency in completing the online questionnaire in either English or Arabic. To identify potential participants, an online questionnaire was distributed through social media channels. Responses from both mothers and fathers of the same adolescent were treated as independent data points.

### 2.3 Data collection

Participants completed a self-administered questionnaire on a specifically designated platform (Google Forms). The questionnaire was re-used from a previous similar study conducted in the United States by Szilagyi et al., after obtaining written permission from the author (14). Some minor modifications were made to a few demographic questions to ensure that the questionnaire was better suited to the Saudi population. The questionnaire was composed of three sections, namely, demographic data (including characteristics of the adolescents and the parents), the validated Vaccine Hesitancy Scale (VHS) (which was originally developed in 2015 by the WHO’s Strategic Advisory Group of Experts [SAGE] on immunization) (16–18), and barriers to receiving the HPV vaccine. The original questionnaire is in English, but the authors (who are all bilingual) undertook a bidirectional translation process to translate the questionnaire to Arabic. Both the Arabic and English versions were distributed online.

### 2.4 Measures

The primary outcome variable for this study was vaccine hesitancy. HPV vaccine hesitancy was defined as a VHS score > 3, since it is the midpoint of the VHS (14). Similar to the previously published US study, we used only 9 of the 10 items in the VHS, because one of the items (All childhood vaccines offered by the government program in my community are beneficial) had previously been considered redundant (14, 19, 20). For each of the 9 questions, we used a 4-point Likert (without neutral responses) to reduce the potential for socially desirable responding. The scale was as follows: 1 = strongly agree, 2 = agree, 4 = disagree, 5 = strongly disagree. Higher scores indicate greater hesitation.

The variables (demographics) considered as predictors of vaccine hesitancy included adolescents’ age and overall health, as well as parents’ demographics (gender, nationality, education level, income, and area of residence) (14, 17).

Finally, six questions examined barriers for HPV vaccination. These included lack of convenience with regard to receiving the HPV vaccine, lack of recommendation of the HPV vaccine by the adolescent’s medical provider, difficulty paying for the vaccine, not having a place to get the vaccine, not having a regular source of care, and lack of HPV vaccine availability at the provider’s office. The respondents responded to all of these six questions with two options—agree and disagree (14, 18).

### 2.5 Statistical analysis

Statistical analysis was completed using IBM SPSS, version 23.0.

Descriptive statistics are presented as frequencies and percentages for the whole study population, and for each of the two groups according to the study’s primary outcome—those who were hesitant versus non-hesitant. The association of each predictor with the outcome (vaccine hesitancy) was assessed using univariate binomial regression for each variable. Then, adjusted analysis was conducted using a multivariate binary logistic regression model. Bivariate Pearson correlation was used to test the assumption of the multivariate binary regression model. Statistical significance was established at *p* < 0.05.

## 3 Results

### 3.1 Demographic variables and evaluation of vaccine hesitancy

**Figure 1:**
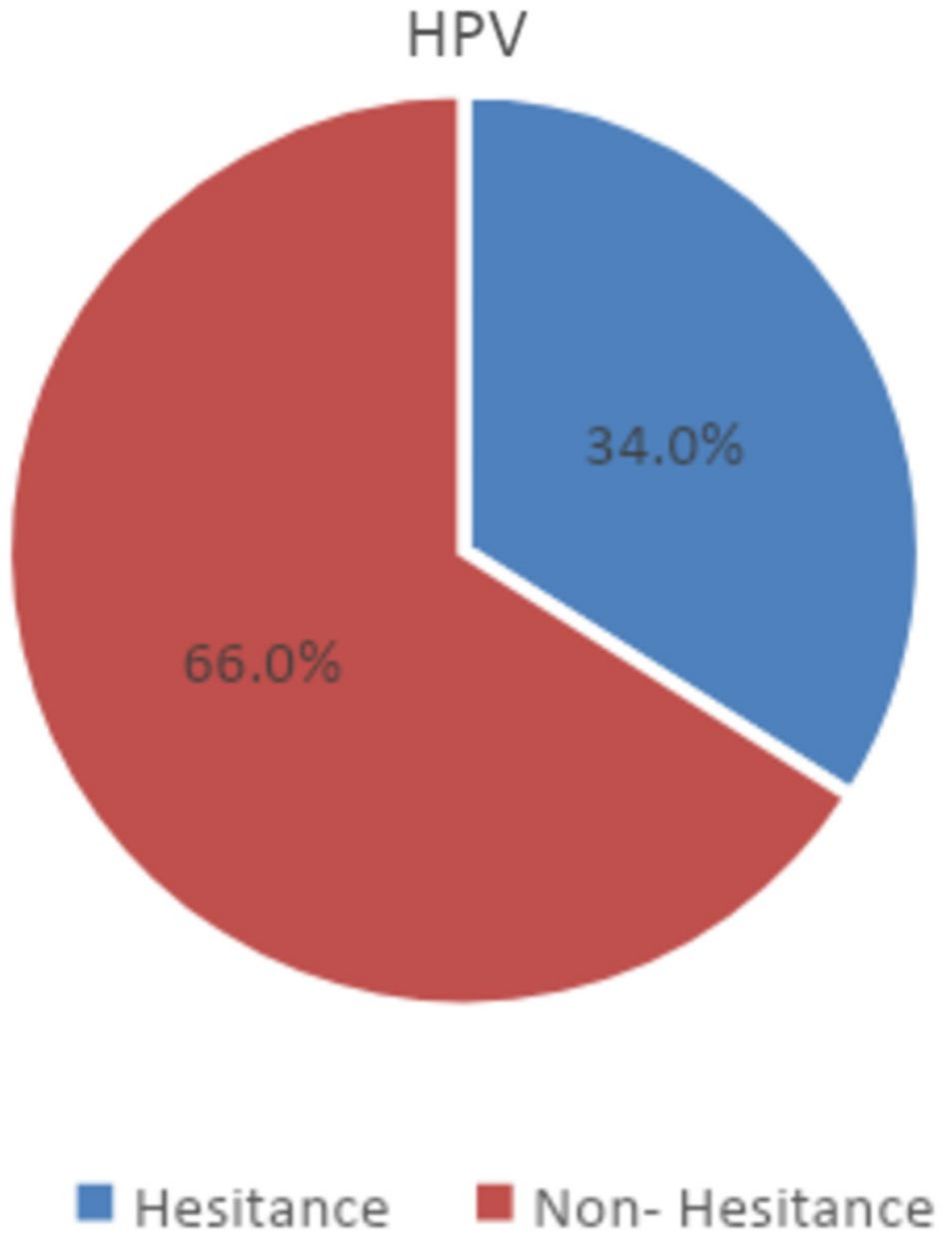
Acceptance of the HPV vaccine among participants (*N* = 667).

Of the 667 participants, 227 (34%) had a VHS score > 3, which signifies vaccine hesitancy, according to our definition. On the other hand, 440 respondents (66%) were non-hesitant based on their VHS score (<3; Figure 1). The majority were parents of adolescents aged 15–17 years (45.4%), as shown in Table 1. Most adolescents, that is, 65.5%, had excellent overall health, and 25.3% were in very good health. Only 14 (2%) displayed weak health, and those with weak health represented 2.6% of the vaccine-hesitant group and 1.8% of the non-hesitant group. Most of the participating parents were females than males (72.5% vs. 27.4%). Further, the female parents represented a greater proportion of the vaccine-hesitant group than the non-hesitant group (76.6% vs. 70.4%). In comparison, male parents formed a greater proportion of the non-hesitant group than of the hesitant group (29.5% vs. 23.3%).

**Table 1:** Demographics of participants (*N* = 667) categorized by vaccine hesitancy.

Participant characteristics		Non-hesitance (*n* = 440)	Hesitance (*n* = 227)	Total (*N* = 667)
Adolescent’s age	11–12 years	138	69	207
		31.4%	30.4%	31.0%
	13–14 years	103	54	157
		23.4%	23.8%	23.5%
	15–17 years	199	104	303
		45.2%	45.8%	45.4%
Adolescent’s overall health	Weak/acceptable	8	6	14
		1.8%	2.6%	2.0%
	Good	31	16	47
		7.0%	7.0%	7.0%
	Very good	118	51	169
		26.8%	22.4%	25.3%
	Excellent	283	154	437
		64.3%	67.8%	65.5%
Gender of parents	Male	130	53	183
		29.5%	23.3%	27.4%
	Female	310	174	484
		70.4%	76.6%	72.5%
Nationality of parents	Saudi	405	210	615
		92.0%	92.5%	92.2%
	Non-Saudi	35	17	52
		7.9%	7.4%	7.7%
Education level of respondent (parent/guardian/foster parent)	High school or less	90	54	144
		20.4%	23.7%	21.5%
	Some college education	57	44	101
		12.9%	19.3%	15.1%
	Bachelor’s degree or higher	293	129	422
		66.5%	56.8%	63.2%
Household income	Less than 4,000 SR	39	40	79
		8.8%	17.6%	11.8%
	4,000–10,000 SR	148	80	228
		33.6%	35.2%	34.1%
	10,000–20,000 SR	178	59	237
		40.4%	25.9%	35.5%
	Greater than 20,000 SR	75	48	123
		17.0%	21.1%	18.4%
Region	East	79	34	113
		17.9%	14.9%	16.9%
	West	189	77	266
		42.9%	33.9%	39.8%
	North	45	17	62
		10.2%	7.4%	9.2%
	South	32	43	75
		7.2%	18.9%	11.2%
	Central Region	95	56	151
		21.5%	24.6%	22.6%

The majority of the participants in our study were Saudi nationals (*n* = 615, 92.2%), had a bachelor’s or higher degree (63.2%), and had a monthly income between 4,000 and 20,000 SR. Detailed baseline characteristics of the respondents are shown in Table 1.

### 3.2 Factors associated with vaccine hesitancy

Table 2 shows the results of unadjusted and adjusted analysis of the predictors of vaccine hesitancy. Each of the demographic variables was tested first by univariate analysis to determine if they were associated with vaccine hesitancy (indicated by a VHS score > 3). Only two variables showed a significant association with vaccine hesitancy—household income and region. Specifically, having a household income of 4,000–10,000 SR or 10,000–20,000 SR was significantly associated with a lower likelihood of vaccine hesitancy than having lower income ranges (odds ratios [OR] for 4,000–10,000 SR = 0.527, *p*-value = 0.015; OR for 10,000–20,000 = 0.32, *p*-value = 0.00). In addition, participants residing in the southern region of Saudi Arabia had higher odds of vaccine hesitancy (OR = 3.122, *p*-value = 0.00) than residents of the Eastern province.

**Table 2:** Unadjusted and adjusted analysis of the variables and their association with HPV vaccine hesitancy (VHS score > 3).

Factor		Unadjusted OR					Adjusted OR				
		*p*-value	Odds ratio (OR)	95% CI for OR		R square	*p*-value	OR	95% CI for OR		R square
				Lower	Upper				Lower	Upper	
Adolescent’s age	11–12 years		*Ref*			0.000		*Ref*			0.093
	13–14 years	0.832	1.049	0.677	1.625		0.664	1.108	0.697	1.764	
	15–17 years	0.817	1.045	0.719	1.519		0.975	1.007	0.671	1.511	
Adolescent’s overall health	Weak/Acceptable		*Ref*			0.004		*Ref*			
	Good	0.548	0.688	0.203	2.327		0.934	0.946	0.251	3.556	
	Very good	0.330	0.576	0.190	1.746		0.948	0.960	0.283	3.260	
	Excellent	0.559	0.726	0.247	2.129		0.657	1.313	0.394	4.374	
Parent’s gender	Male		*Ref*			0.006		*Ref*			
	Female	0.090	1.377	0.951	1.992		0.453	1.165	0.782	1.734	
Parent’s nationality	Saudi		*Ref*			0.000		*Ref*			
	Non-Saudi	0.832	0.937	0.513	1.712		0.269	0.686	0.351	1.339	
Parent education	High school or less		*Ref*			0.014		*Ref*			
	Some college education	0.341	1.287	0.766	2.160		0.287	1.354	0.775	2.368	
	Bachelor’s degree or higher	0.125	0.734	0.494	1.090		0.683	0.912	0.586	1.420	
Household income	Less than 4,000 SR		*Ref*			0.041		*Ref*			
	4,000–10,000 SR	**0.015**	0.527	0.314	0.885		**0.023**	0.526	0.303	0.915	
	10,000–20,000 SR	**0.000**	0.323	0.190	0.549		**0.000**	0.331	0.182	0.600	
	Greater than 20,000 SR	0.105	0.624	0.353	1.104		0.099	0.583	0.308	1.106	
Region	East		*Ref*			0.046					
	West	0.823	0.947	0.585	1.532		0.733	0.916	0.555	1.514	
	North	0.710	0.878	0.441	1.746		0.492	0.777	0.379	1.595	
	South	**0.000**	3.122	1.698	5.741		**0.004**	2.605	1.355	5.007	
	Central Region	0.236	1.370	0.814	2.304		0.341	1.300	0.758	2.230	

Bold values are statistically significant.

To adjust for potential confounders among the predictor variables, adjusted analysis was conducted using the multivariable logistic regression model (Table 2). In this analysis, too, household income and region emerged as significant variables. As observed in the univariate analysis, the household income categories 4,000–10,000 SR and 10,000–20,000 SR were significantly associated with a lower likelihood of vaccine hesitancy than the lower income categories (OR for 4,000–10,000 = 0.526, *p*-value = 0.023; OR for 10,000–20,000 = 0.33, *p*-value = 0.00). Further, participants residing in the southern region of Saudi Arabia had higher odds of vaccine hesitancy (OR = 2.605, *p*-value = 0.004) than residents of the Eastern province. None of the other demographic variables were significantly associated with vaccine hesitancy (Table 2).

**Figure 2:**
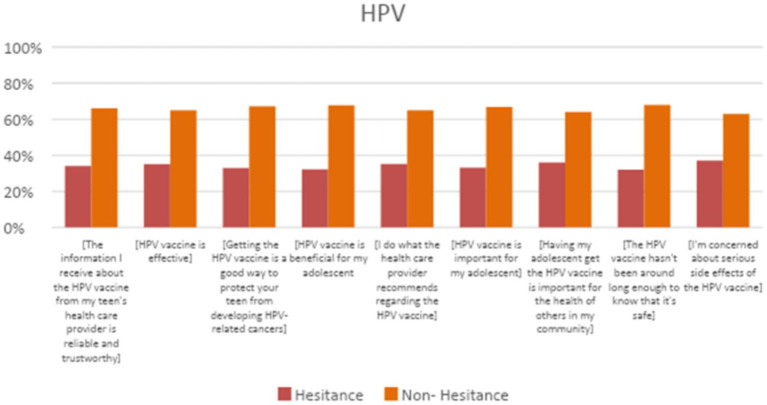
Parental perceptions about HPV vaccination items from the 9 item modified VHS scale were adapted for the HPV vaccine.

**Figure 3:**
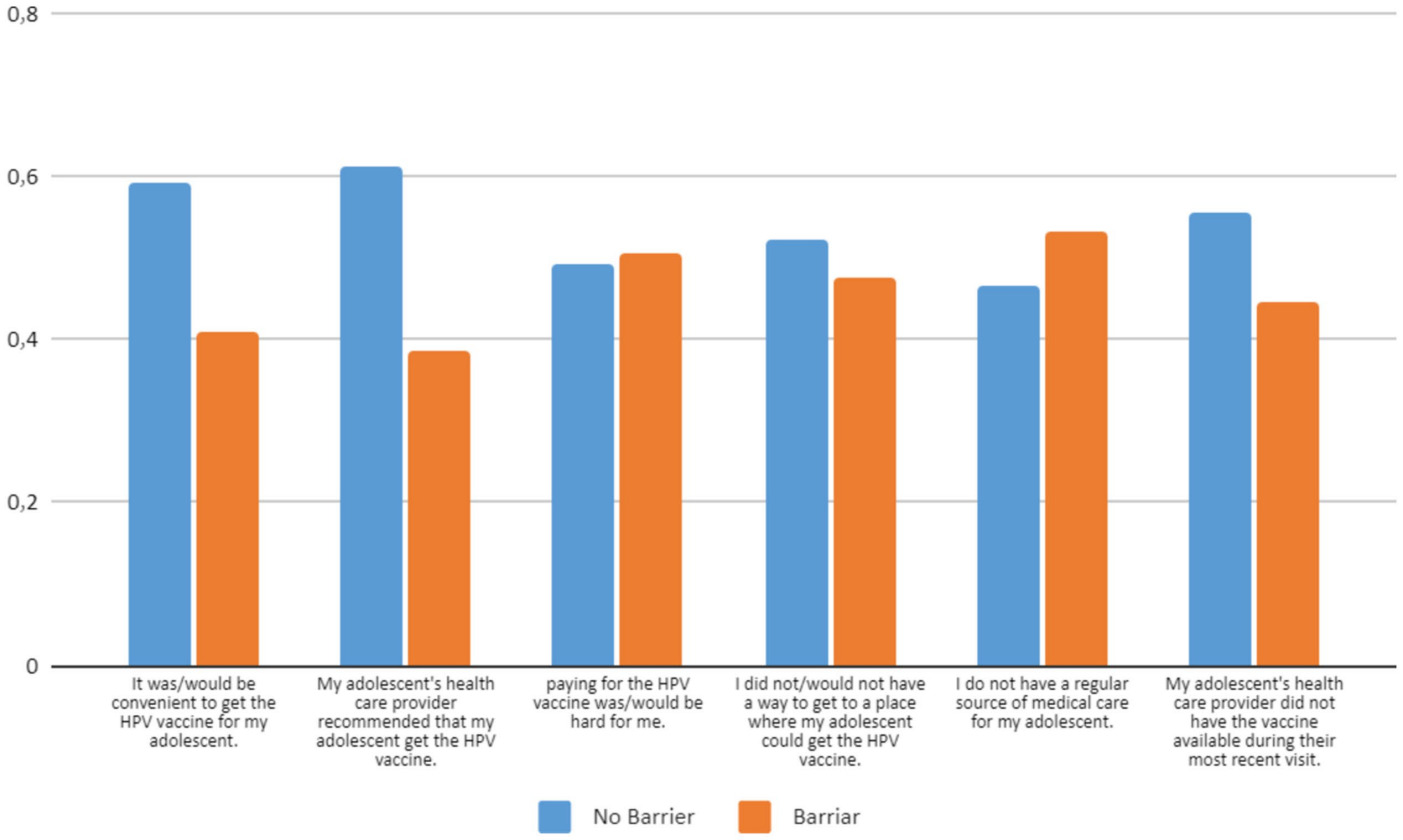
Parents’ responses with regard to barriers to immunizing their adolescent female children with the HPV vaccine.

Figure 2 shows parental responses to the 9-item modified VHS scale, that was adapted for the HPV vaccine. Figure 3 shows the participants’ responses to questions regarding potential barriers to receiving the HPV vaccine. For each question, respondents had the option to respond with “agree” or “disagree.” Their responses were used to classify each variable as being “a barrier” or “not a barrier” to receiving the HPV vaccine (Figure 3). The most common variables found to be barriers were not having a regular source of medical care for the adolescent (53.4%), difficulty paying for the HPV vaccine (50.7%), and finding transport to a place that provides the HPV vaccine (47.7%). The rest of the barriers are shown in Figure 3.

### 3.3 Significant barriers to vaccine hesitancy

Table 3 shows the results of unadjusted and adjusted analysis of the barriers associated with vaccine hesitancy. Each of the variables was tested first by univariate analysis for association with vaccine hesitancy (indicated by a VHS score > 3). Three barriers showed a significant association with vaccine hesitancy: convenience of receiving the vaccine (OR = 3.941, *p* = 0.001), lack of recommendation by the adolescent’s health care provider (OR = 4.811, *p* = 0.001), and difficulty finding a place to receive the vaccine (OR = 0.594, *p* = 0.002). These three variables remained significantly associated with vaccine hesitancy after adjusted analysis: convenience of receiving the vaccine (OR = 2.141, *p* = 0.001), lack of recommendation by the adolescent’s health care provider (OR = 2.882, *p* = 0.000), and difficulty finding a place to receive the vaccine (OR = 0.498, *p* = 0.003).

**Table 3:** Practical barriers to receiving the HPV vaccine and their relationship to HPV vaccine hesitancy (*N* = 667).

Factor		Unadjusted OR					Adjusted OR				
		*p*-value	OR	95% CI for OR		R square	*p*-value	OR	95% CI for OR		R square
				Lower	Upper				Lower	Upper	
It was/would be convenient to get the HPV vaccine for my adolescent.	No Barrier		*Ref*			0.131		*Ref*			0.200
	Barrier	**0.001**	3.941	2.811	5.523		**0.001**	2.141	1.381	3.320	
My adolescent’s health care provider recommended that my adolescent get the HPV vaccine.	No Barrier		*Ref*			0.166		*Ref*			
	Barrier	**0.001**	4.811	3.413	6.781		**0.000**	2.882	1.859	4.469	
Paying for the HPV vaccine was/would be hard for me.	No Barrier		*Ref*			0.006		*Ref*			
	Barrier	0.101	0.765	0.555	1.054		0.515	1.139	0.770	1.685	
I did not/would not have a way to get to a place where my adolescent could get the HPV vaccine.	No Barrier		*Ref*			0.021		*Ref*			
	Barrier	**0.002**	0.594	0.429	0.823		**0.003**	0.498	0.316	0.786	
I do not have a regular source of medical care for my adolescent.	No Barrier		*Ref*			0.000		*Ref*			
	Barrier	0.850	0.969	0.703	1.336		0.889	1.030	0.676	1.570	
My adolescent’s health care provider did not have the vaccine available during the most recent visit.	No Barrier		*Ref*			0.000		*Ref*			
	Barrier	0.879	1.025	0.743	1.415		0.128	1.408	0.906	2.187	

Bold values are statistically significant.

## 4 Discussion

The present study investigated the level of hesitancy among parents to get their adolescent children immunized with the HPV vaccine in Saudi Arabia. We found that 34.3% of the participating Saudi parents of adolescents (between 11 to 17 years old) were hesitant about immunization their children with the HPV vaccine. A previous similar study conducted in the western region of Saudi Arabia concluded that parents had a poor level of knowledge about HPV infection and its vaccine and only 7.2% of them had vaccinated their female children (15). Another study among adult women in Saudi Arabia (2021) found that about 55% would accept the HPV vaccine if offered and about 73% were willing to advice others to take the vaccine (21). The present findings are relevant because they provide a national-level picture of the trends in HPV vaccine hesitancy among parents of adolescents. We believe the findings will help establish vaccine awareness and acceptance policies and strategies at the national level.

We examined the factors associated with vaccine hesitancy and found that lower monthly income was a predictor of HPV vaccine hesitancy. This finding is consistent with some previous studies conducted in other countries. For example, in 2019, a US study indicated that higher-income families are less hesitant about the vaccine (14), while a study from China found that women with higher household income were more likely to receive the HPV vaccine (22). This may be due to better access to vaccine-related resources, education, and affordability among higher-income families. In contrast, a study from the 2010 US National Immunization Survey-Teen Survey found that families with greater incomes were more likely to be reluctant to receive the HPV vaccine (23).

Our results show that there was no relationship between parents’ educational level and hesitancy to take the vaccine. This might be attributed to Saudi Arabian government’s efforts toward raising awareness about the vaccines and establishing several primary health care centers that provide easy access to various vaccines (10). These efforts might have ensured that the information and vaccine are accessible to people of all backgrounds. On the other hand, a study done in France found that hesitancy to receive HPV was high among educated parents (24).

In this study, we observed significant regional differences in vaccine hesitancy. Specifically, parents from the southern region were more hesitant about the vaccine compared to those from other areas. A similar study conducted in Jazan city, also in the south, found a notably low acceptance level for the HPV vaccine (4). These regional disparities may stem from more conservative cultural beliefs in certain areas. For instance, individuals in the southern and northern regions may adhere to more conservative values than those in urban centers in the middle, eastern, and western regions of Saudi Arabia. Hesitancy in these conservative cultures may be linked to the perception that the HPV vaccine encourages earlier sexual activity.

With regard to the reasons for vaccine hesitancy, our study found that many parents do not believe the HPV vaccine is actually helpful for their adolescent child or that it protects them against HPV-related cancers. Some others were concerned about the safety of the vaccine given that it is new. Similarly, in a study conducted in Jazan city, concerns about the safety, efficacy, and novelty of the HPV vaccine were cited as reasons for hesitancy. In addition, there was some concern that the vaccine would lead to earlier sexual conduct (4). In the present study, around a third (34%) of our respondents expressed that they do not trust the information about the HPV vaccine that they received from their adolescent’s healthcare provider. This reason was also cited in a previous U.S study, which reported the parentage of respondents with this issue to be around 20%. However, when looking at barriers and their association with HPV-vaccine hesitancy, not having the recommendation of the healthcare provider seems to be significantly associated with vaccine hesitancy. In addition, parents who found it convenient for their adolescent to receive the HPV vaccine were less hesitant to receive the vaccine. A comparable analysis conducted in 2020 by Peter Szilagyi yielded similar results: that is, parents were less apprehensive if it was convenient to immunize their adolescent or if their doctor had advised the adolescent to receive the HPV vaccine (14). Some research at the national level in Saudi Arabia has assessed the reasons why people are hesitant about receiving the HPV vaccine (25, 26).

In line with our findings, the Centre for Disease Control and other experts on vaccines have emphasized the value of strong and persuasive provider recommendation for the HPV vaccine as well as the crucial role that clinicians play in parents’ decisions to accept or reject the vaccine (27, 28). Only 61% of parents in this study agreed that their adolescent’s primary care physician had recommended the HPV vaccine. However, it is important to keep in mind that recall bias may affect parents’ memories of a strong physician recommendation. For example, cautious parents may be more inclined to claim their provider did not prescribe the immunization. Unfortunately, there is no simple way to connect parental memories of provider communications about the HPV vaccine with observations of provider communications about the HPV vaccination, and there is no research to our knowledge that addresses this issue. Irrespective of this, it is important that providers are aware of parents’ hesitation and are able to address specific parental concerns about the HPV vaccine. Accordingly, specialists have started researching ways to speak to parents and teenagers about HPV vaccine in a more effective manner, and some promising results are emerging in this area (27, 29–32). However, for physicians and practices to best support parents in their decisions about HPV vaccination, much more work has to be done to develop and implement scalable strategies that are practical within relatively constrained office visit durations.

Our study has some limitations that need to be borne in mind while interpreting the results. The first limitation is that the sample might not be representative of the entire Saudi population. Thus, in the future, larger population-based studies looking at the prevalence of vaccine hesitancy and utilization are needed. As with all observational studies, this analysis also showed associations for which causality cannot be established.

## 5 Conclusion

HPV vaccine hesitancy among parents of adolescent females is still high in Saudi Arabia, with around one-third of the participating parents expressing hesitancy. Based on our analysis, it seems that lower income and living in the southern region may be associated with higher vaccine hesitancy. In addition, inconvenience related to receiving the vaccine and not having the recommendation from the healthcare providers are also associated with higher vaccine hesitancy. More efforts from physicians to educate parents and adolescents, as well as making the vaccination available and convenient, are needed to raise the acceptance levels.

## Data Availability

The original contributions presented in the study are included in the article/supplementary material, further inquiries can be directed to the corresponding author.
